# Development of a photometric system for continuous flow analysis

**DOI:** 10.1155/S1463924603000087

**Published:** 2003

**Authors:** S. S. Randhawa, Baban K. S. Bansod, Anirudh K. Singh, G. Chand, A. K. Ganju

**Affiliations:** Central Scientific Instruments Organisation Chandigarh 160030 India

## Abstract

Most chemical analyses carried out in a clinical laboratory are colorimetric. An improved photometric system is described where a tungsten lamp is the light source, a photo-diode is the detector and a microcontroller 8051 is used for processing and displaying absorbances. The performance characteristics of the instrument are reported. The parameters investigated are photometric linearity, precision and instrumental drift.


Journal of Automated Methods & Management in Chemistry
Vol. 25, No. 2 (March–April 2003) pp. 51–55

Taylor & Francis

Taylor & Francis Group

### Development of a photometric system for continuous flow analysis

**S. S. Randhawa*, Baban K. S. Bansod, Anirudh K. Singh, G. Chand and A. K. Ganju**

*Central Scientific Instruments Organisation, Chandigarh—160030, India*

*Most chemical analyses carried out in a clinical laboratory are colorimetric. An improved photometric system is described where a tungsten lamp is the light source, a photo-diode is the detector and a microcontroller 8051 is used for processing and displaying absorbances. The performance characteristics of the instrument are reported. The parameters investigated are photometric linearity, precision and instrumental drift.*

### Introduction

The development and use of chemicals in agriculture, animal husbandry, and human health and nutrition have increased the awareness of the importance of the chemical environment and the effects of synthetic compounds on the flora and fauna. While modern research and service-oriented laboratories of industrialized nations increasingly use sophisticated instruments, there is a growing need for compact, inexpensive and portable instruments that can perform determinations in regions far from the convenience of a modern laboratory [1]. Recent advances in low-cost, miniaturized detection technology [2–4] have made it possible to take traditional laboratory-based instrumental techniques, e.g. a UV/visible spectrophotometer, to remote sites for *in-situ* monitoring. In flow spectrophotometry, multiple measurements on a processed sample are accomplished efficiently in order to permit implementation of simultaneous determinations, differential kinetic analysis, speciation, standard addition and blank compensation [5–8]. The present paper describes a brief performance evaluation of the photometric system that can be used for the continuous flow analysis of the determination of various parameters in clinical, environmental and food chemistry.

### Experimental

Solid potassium permanganate was dried for 1 h at 100 °C. A stock solution was prepared by dissolving potassium permanganate 0.2 g in 1 litre distilled water. A set of standards (0.5, 1.0 and 1.5 ml of stock solution diluted up to 4 ml with distilled water, known as I, II and III standards, respectively) was prepared by serial dilution of the stock solution. A 10% cobalt chloride solution was prepared by dissolving cobalt chloride in distilled water. These standards were analyzed immediately after preparation.

* To whom correspondence should be addressed.
e-mail: ss_randhawa1@rediffmail.com

### *Instrumentation*

A block diagram of the instrument is shown in figure 1. The instrument consists of a light source, a focusing lens, filters, photodetectors and a readout system. The tungsten lamp was used as a light source and emitted radiation in the visible wavelength region. The photodiode used as a detector was placed on the opposite side of a flow cell of 10-mm path length. Source stability was achieved by a constant voltage transformer and an electronic regulator. The transmittance signals were fed through a preamplifier, a log amplifier and through an A/D converter to an 8051 microcontroller for processing and display of the absorbance.

The lamp was allowed to warm up for 10 min before use. The system was designed to allow different modes of operation. The first operating mode was to check the zero position. It then calibrated to 100% transmission (T) with distilled water at $\lambda_{max}$. The next operating mode was to flow the standard concentration solution or for a particular solution of known absorbance.

Absorbance was calculated according to following equation:

$$A = \log[(I_0 - I_d)/(I - I_d)],$$

where $A$ is the absorbance for a particular wavelength in a given flow cell, $I_d$ is the dark current, and $I_0$ and $I$ are the digital signals (proportional to the light intensity) observed while the blank solution and sample solution are flowed through the flow cell, respectively.

### Results and discussion

Potassium dichromate (0.005 M) is a commonly used solution for the routine calibration of spectrophotometer [9] and is readily available from commercial suppliers as a calibration set. However, these are used in the 200–400 nm region and therefore require a UV source. Potassium permanganate was therefore chosen as the standard for the visible region due to its well-defined absorbance spectrum [10].

Spectrophotometric methods usually require the isolation of discrete bands of radiation. Beer's law, which is the

*Journal of Automated Methods & Management in Chemistry* ISSN 1463–9246 print/ISSN 1464–5068 online © 2003 Taylor & Francis Ltd
http://www.tandf.co.uk/journals
DOI: 10.1080/1463924031000139329

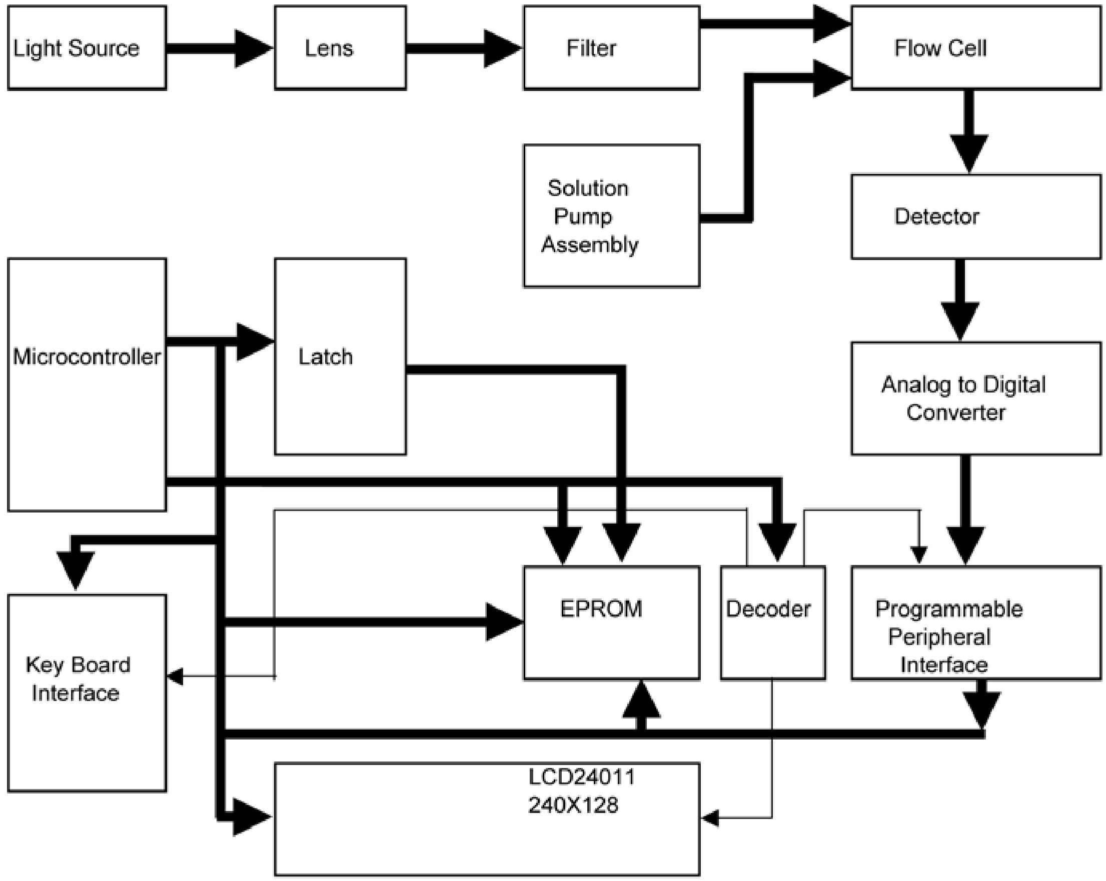

*Figure 1. Block diagram of the photometric system.*

basis of quantitative work, is based on the assumption of monochromatic radiation. Additionally, in an emission mode, the most favourable signal ratio between the background and the analytical emission lines must be selected. To isolate a narrow band of wavelengths, filters or monochromators are used. However, we used the interference filters in the improved photometric system. These are optical filters that remove unwanted wavelengths of light by interference phenomena rather than by absorbance or scattering. They can be made to transmit a very narrow band of wavelengths. This type of filter is composed of an externally thin dielectric layer sandwiched between two semireflecting metallic films. The characteristic curve of the interference filter is shown in figure 2.

The system had a dual optical system. Both the reference and sample beam emanate from a single source. The reference beam passes through a lens and a filter before reaching its detector. The sample beam passes through a flow cell before reaching the sample photodetector. The flow cell used in the sample channel was constructed from a glass tube with an inlet, a debubbler and a waste outlet. The mounting of the flow cell was such that it permitted slight adjustment for proper alignment of the optical beam and flow stream.

The solution was continuously pushed through the flow cell by the pumping system, which consisted of a roller head assembly, a pulley with a belt and induction motor

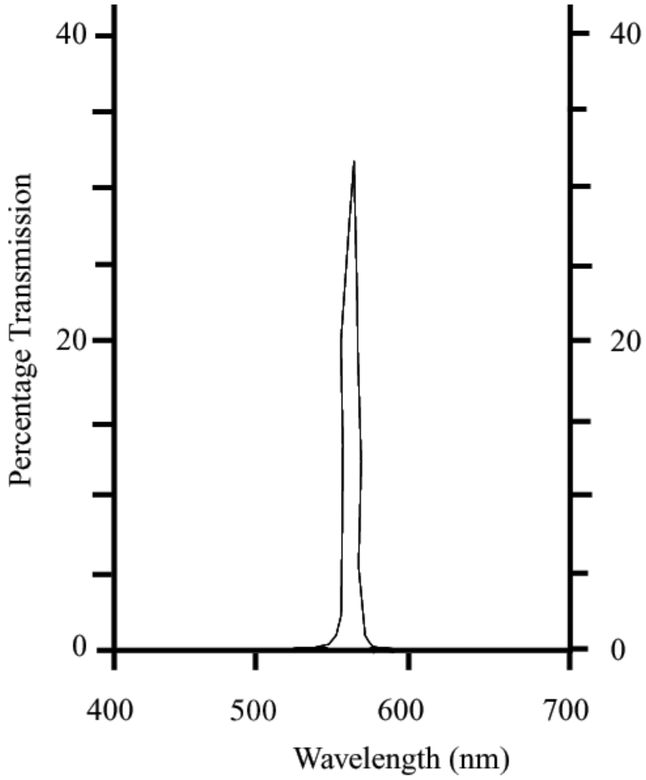

*Figure 2. Characteristic curve of the interference filter used.*

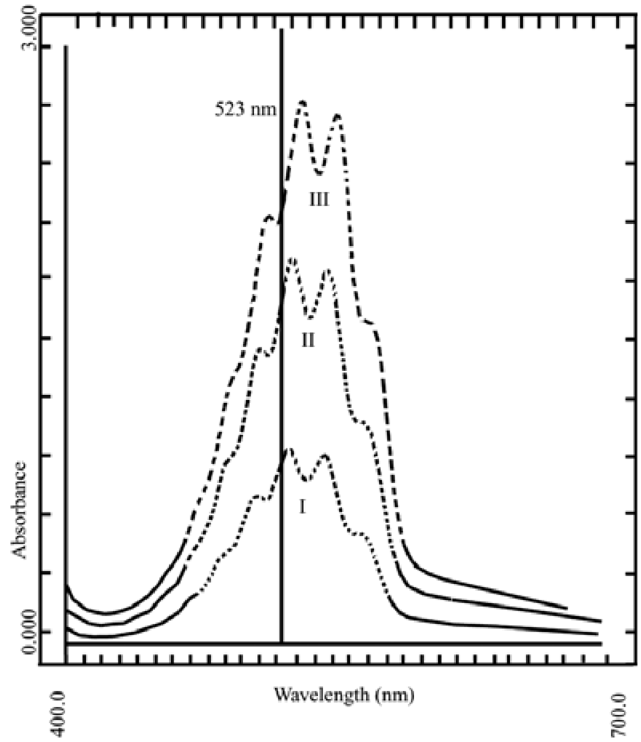

*Figure 3. Spectra of potassium permanganate standards.*

(figure 4). As the roller head assembly moves in a circular path, the tube is pressed and the solution is pulled from the bottle and pushed through the flow cell at a constant speed.

The 8051 microcontroller used to process and display the absorbance of the base line, reference and sample absorbance had an 8-bit CPU with accumulator A and B registers, a 16-bit program counter and data pointer, an 8-bit stack pointer, an internal EPROM of 4 k, an internal RAM of 128 bytes, 32 I/O pins arranged as four 8-bit port, two 16-bit timer/counter, full duplex serial data transmitter/receiver, and control registers. It has external and internal interrupt sources, an oscillator and clock circuits. The output of a detector is given to an analogue-to-digital (ADC 7109) converter. Port A of 8255 was used to read the ADC, port c of 8255 was used to control the signal of 7109. As the program was loaded in EPROM, the 8051 microcontroller started the program execution from EPROM.

The instrumental drift is defined as long-term (hours) variation in measured absorbance. Drift should be very low with double or split-beam spectrophotometers because they correct this problem inherently. However, for single-beam instruments, the drift is important because it is an indicator of how frequently a blank measurement must be made to ensure a desired accuracy. Tables 1 and 2 show the results obtained with the improved photometer system using a potassium permanganate standard monitored at $\lambda_{max}$ for 80 min. The sampling period was divided into eight time slices as in table 1 and four time slices in table 2. Maximum and minimum absorbances during a time slice, and positive and negative deviation from the mean absorbance are also shown. The overall drift of 0.018 a.u. $h^{-1}$ was found for the instrument. Ten readings of the potassium permanganate standard II and stock solution were taken every 1 min. These reading were then averaged to give a mean absorbance. The maximum mean absorbance of 0.201 a.u. was in the 30–40-min time slice and lowest value of 0.181 was found in the 71–80-min time slice in the standard II solution. However, in the case of

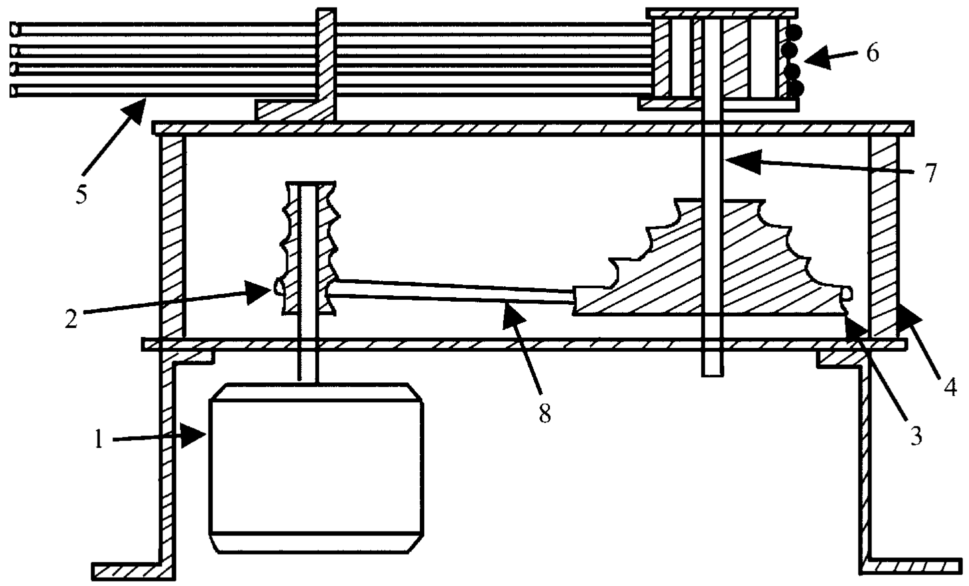

*Figure 4. Solution pumping assembly. 1, Motor; 2, motor pulley; 3, speed control pulley; 4, frame; 5, sample plastic tube; 6, roller assembly; 7, shaft; 8, belt.*

*Table 1. Instrumental drift for potassium permanganate standard II monitored at $\lambda = 558$ nm.*

| Time slice (min) | Mean absorbance (a.u.) | Standard deviation (a.u.) | Maximum (a.u.) | Maximum positive deviation from mean (a.u.) | Minimum (a.u.) | Maximum negative deviation from mean (a.u.) |
|---|---|---|---|---|---|---|
| 00–10 | 0.192 | 0.003 | 0.197 | 0.005 | 0.186 | 0.006 |
| 11–20 | 0.195 | 0.005 | 0.201 | 0.006 | 0.192 | 0.003 |
| 21–30 | 0.198 | 0.004 | 0.203 | 0.005 | 0.188 | 0.010 |
| 31–40 | 0.201 | 0.006 | 0.203 | 0.002 | 0.197 | 0.004 |
| 41–50 | 0.194 | 0.004 | 0.198 | 0.004 | 0.187 | 0.007 |
| 51–60 | 0.192 | 0.003 | 0.197 | 0.005 | 0.187 | 0.005 |
| 61–70 | 0.185 | 0.003 | 0.191 | 0.006 | 0.180 | 0.005 |
| 71–80 | 0.181 | 0.003 | 0.184 | 0.003 | 0.179 | 0.002 |

*Table 2. Instrumental drift for potassium permanganate stock solution monitored at $\lambda = 558$ nm.*

| Time slice (min) | Mean absorbance (a.u.) | Standard deviation (a.u.) | Maximum (a.u.) | Maximum positive deviation from mean (a.u.) | Minimum (a.u.) | Maximum negative deviation from mean (a.u.) |
|---|---|---|---|---|---|---|
| 00–10 | 0.639 | 0.003 | 0.644 | 0.005 | 0.634 | 0.005 |
| 11–20 | 0.636 | 0.006 | 0.647 | 0.011 | 0.630 | 0.006 |
| 21–30 | 0.626 | 0.004 | 0.637 | 0.011 | 0.621 | 0.005 |
| 31–40 | 0.624 | 0.003 | 0.631 | 0.007 | 0.620 | 0.004 |

*Table 3. Photometric accuracy for different solutions of cobalt chloride at $\lambda = 558$ nm.*

| Percentage of cobalt chloride solution | Absorbance measured with a DIGISPEC spectrophotometer ($n = 10$) | Mean absorbance with the developed instrument ($n = 10$) | Difference in the two mean absorbances (a.u.) | Percentage of coefficient of variation in the developed instrument |
|---|---|---|---|---|
| 2.5 | 0.144 | 0.159 | 0.015 | 1.25 |
| 5.0 | 0.173 | 0.191 | 0.018 | 1.04 |
| 7.5 | 0.210 | 0.220 | 0.010 | 0.90 |
| 10 | 0.210 | 0.222 | 0.012 | 0.45 |

stock solution reading, the maximum mean absorbance 0.639 a.u. was in the 0–10-min time slice. The absorbances of potassium permanganate standard solutions measured with the developed photometric system were slightly lower than the expected values. This may be due to the filter having a higher wavelength and to the non-availability of the exact wavelength filter.

Figure 3 shows the spectrum for permanganate standards with different absorbance units, which were recorded with an ELICO UV-VIS Spectrophotometer in the wavelength range 400–700 nm. Table 3 shows the photometric accuracy of the results. The absorbances of the cobalt chloride solution were also compared with the DIGISPEC spectrophotometer (table 3). The maximum coefficient of the variation in the developed instrument was 1.25% in the 2.5% solution and had a minimum of 0.45% in the 10% cobalt chloride solution.

### Conclusion

The flow type photometric system is very low cost in comparison with a commercial-type spectrophotometer, is inherently portable and had a rugged construction, all of which make it ideally suited for the field and other process developments. Instrument linearity and photometric precision are sufficiently good for its use in long-term operations.

### Acknowledgements

The authors thank the Director of the Central Scientific Instruments Organisation, Chandigarh, for encouragement, guidance and permission to publish the paper. This programme was funded by the Technical Mission on Oil Seeds, Pulses and Maize (TMOP&M), Ministry of Agriculture, Government of India, New Delhi. A. K. S. was a 'Project Assistant' under TMOP&M programme.

### References

1. Kraus, P. R., Wade, A. P., Crouch, S. R., Holland, J. F. and Miller, B. M., *Analytical Chemistry*, **60** (1988), 1387.
2. Mazel, C. H., *Optical Engineering*, **36** (1997), 2612.
3. Belz, M., Boyle, W. J. O., Klein, K. F. and Grattan, K. T. V., *Sensors and Actuators B*, **39** (1997), 380.
4. Coles, S., Nimmo, M. and Worsfold, P. J., *Journal of Automated Methods & Management in Chemistry*, **22** (2000), 97.
5. Randhawa, S. S., Gupta, R. C., Bhandari, A. K. and Malhotra, P. S., *Journal of Automated Chemistry*, **14** (1992), 185.
6. Santos, S. R. B., Araujo, M. C. U., Honorato, R. S., Zagatto, E. A. G., Lima, J. F. C. and Lapa, R. A. S., *Journal of Automated Methods & Management in Chemistry*, **22** (2000), 83.
7. Randhawa, S. S., Chand, G. and Ganju, A. K., *Journal of Automated Method & Management in Chemistry*, **24** (2002), 49.
8. Hansen, E. H., Muller, H. and Muller, V., *Analytica Chimica Acta*, **230** (1990), 113.
9. Burgess, C. and Knowles, A. (eds), *Standards in Absorption Spectrometry* (London: Chapman & Hall, 1981).
10. Thomas, M., *Ultraviolet and Visible Spectroscopy* (Chichester: Wiley, 1996).

